# Supplementary material for: Impaction Predictors and Diagnostic Performance of CBCT Versus Panoramic Radiography for Supernumerary Teeth in a Romanian Multicenter Cohort
Source: Diagnostics (Basel). 2025 Nov 27;15(23):3019. doi: 10.3390/diagnostics15233019 (PMC12691520; doi:10.3390/diagnostics15233019)
Supplement: Supplementary file 1 [file diagnostics-15-03019-s001.zip › diagnostics-3983915-supplementary.pdf]

Supplementary Materials

Table S1. Multivariable logistic regression model and sensitivity analyses evaluating predictors of impaction in supernumerary teeth (n = 153)

Predictor	Adjusted OR	95% CI	p-value	VIF
Age < 13 years	2.21	1.08–4.52	0.030	1.32
Sex (Male)	1.41	0.74–2.70	0.291	1.06
Morphology – Tuberculate	3.94	1.92–8.06	<0.001	1.87
Morphology – Supplemental	1.32	0.67–2.58	0.411	1.22
Location – Lateral	2.88	1.42–5.81	0.003	1.45
Angle Class III	3.17	1.26–7.93	0.014	1.51
Constant	—	—	0.022	—

Statistic	Value
Hosmer–Lemeshow test (p-value)	0.48
AUC (ROC curve)	0.79
Range of VIF values	1.06–1.87
Missing data (%)	<2%
Missing data strategy	Complete-case + multiple imputation (m = 5)

Sensitivity Analyses

1. Age as a continuous predictor

Predictor	Adjusted OR	95% CI	p-value
Age (continuous)	0.93	0.87–0.99	0.032

2. Alternative age cut-off: <12 years

Predictor	Adjusted OR	95% CI	p-value
Age < 12 years	2.05	1.03–4.10	0.040

3. Alternative age cut-off: <10 years

Predictor	Adjusted OR	95% CI	p-value
Age < 10 years	1.74	0.82–3.70	0.147

Table S2. Diagnostic performance of OPG compared with CBCT for detecting impacted and laterally positioned supernumerary teeth (n = 185 teeth)

**CBCT (Reference Standard)**	**Impacted / Lateral (Positive)**	**Not Impacted (Negative)**	**Total**
**OPG Positive**	56 (True Positive)	8 (False Positive)	64
**OPG Negative**	14 (False Negative)	107 (True Negative)	121
**Total**	70	115	185

Note: Percentages refer to the number of supernumerary teeth (n = 185).

Diagnostic Performance Metrics

- Sensitivity: 56 / 70 = 80.0%
- Specificity: 107 / 115 = 93.0%
- Positive Predictive Value (PPV): 56 / 64 = 87.5%
- Negative Predictive Value (NPV): 107 / 121 = 88.4%
- Overall Accuracy: (56 + 107) / 185 = 88.1%

Interpretation:

OPG shows high specificity and good sensitivity, indicating solid performance in identifying non-impacted ST and a reasonable ability to detect impacted/lateral ST. False negatives occur mainly in laterally displaced or tuberculate teeth, supporting selective CBCT use in these presentations.

Figure S1. ROC Curve

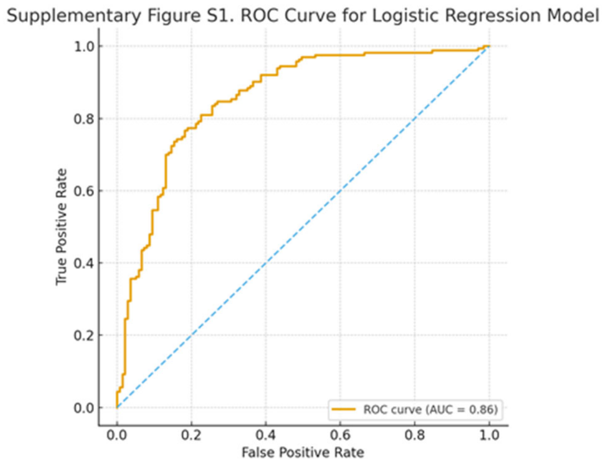

Figure S1. ROC curve for the logistic regression model predicting impaction in supernumerary teeth.

The model demonstrates acceptable discrimination, with an AUC of 0.79, consistent with the diagnostic capacity expected for morphological and positional predictors evaluated in this study.
